# Polyethylene Glycol Diacrylate Adapted Photopolymerization Material for Contact Lens with Improved Elastic Modulus Properties

**DOI:** 10.3390/ma18040827

**Published:** 2025-02-13

**Authors:** Yamin Chen, Dianyang Li, Yougen Chen, Hui Fang

**Affiliations:** 1 Nanophotonics Research Center, Shenzhen Key Laboratory of Micro-Scale Optical Information Technology, Institute of Microscale Optoelectronics, Shenzhen 518060, China; 2200493017@email.szu.edu.cn (Y.C.); lidianyang2022@email.szu.edu.cn (D.L.); 2 Institute for Advanced Study, Shenzhen University, Shenzhen 518060, China

**Keywords:** PEGDA, silicone hydrogel material, contact lens, elastic modulus, Zemax simulation

## Abstract

Four kinds of silicone hydrogel transparent contact lenses (CLs) with different formulations were prepared by the free radical photocuring polymerization. By mixing polyethylene glycol diacrylate (PEGDA) of 1000 Da with ethylene glycol dimethacrylate (EGDMA) and adding other silicone monomers and hydrophilic monomers, the transparency and flexibility of the material were successfully achieved. By optimizing the weight percentage of each component, the best balance of optical performance can be achieved. The photocuring properties of the materials were characterized by electronic universal test, double-beam UV-visible spectrophotometer, Atomic Force Microscope (AFM), Scanning Electron Microscope (SEM) and Fourier Transform Infrared Spectroscopy (FTIR). The results showed that the addition of higher PEGDA content reduces the elastic modulus, improves curing efficiency, improves equilibrium water content (EWC), and enhances light transmission. Hydrogels containing only high PEGDA but no EGDMA showed similar curing rates, water content, and elastic modulus, but had the worst optical transparency, far inferior to the materials mixed with PEGDA and EGDMA. Additionally, imaging performance of the CLs was further evaluated through simulation analysis using Ansys Zemax OpticStudio2024 software. This research provides a new choice of material consideration to improve the performance and wearing comfort of CLs.

## 1. Introduction

**Figure 1:**
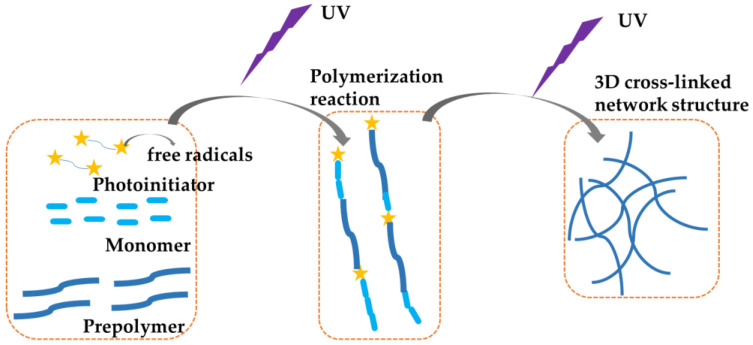
Principle of photocuring free radical polymerization.

Contact lenses (CLs) play an important role in the modern optical material technology by acting as an important vision correction tool. Currently, CLs are predominantly prepared via free radical polymerization photocuring [1]. This photocurable free radical polymerization technology operates on a chain-growth polymerization mechanism. As illustrated in Figure 1, its principle involves the steps that the photoinitiators absorb the energy of the ultraviolet light source after which the chemical bond breaks and free radicals are produced. Then these active free radicals are polymerized and cross-linked with oligomeric monomers, and finally the liquid mixture is transformed into a solid material. Depending on the different preparation materials, CLs are mainly categorized into soft (manufactured from hydrogel material), Rigid Gas Permeable (RGP), and hybrid (featuring a central optical zone made of RGP material, surrounded by a peripheral fitting zone composed of a silicone hydrogel) [2].

We center our research on soft CLs, which are typically composed of hydrogel or silicone hydrogel. Compared with rigid CLs, they offer greater comfort and flexibility. Today, the majority of CLs in use are synthesized through the polymerization of two or more monomers. This enables the polymers to integrate the properties of individual monomers [3,4,5,6], and this approach serves as a guiding principle for the development of CLs materials [1]. CLs made from traditional hydrogel materials such as 2-hydroxyethyl methacrylate (HEMA) and 1-vinyl-2-pyrrolidinone (NVP) are generally very hydrophilic. However, their oxygen permeability is often insufficient for long-term wear, especially when the eyes are closed [7]. In recent years, silicone monomers with strong hydrophobic properties, such as 3-(methacryloyloxy) propyltris (trimethylsiloxy) silane (TRIS), have been introduced to significantly improve the oxygen permeability of CLs [8,9]. Nonetheless, this improvement frequently brings about discomfort and dryness for the wearer, thereby restricting their widespread application [10,11]. To address this issue, gamma-methacryloxypropyltrimethoxysilane (KH-570), a bifunctional organic silicone monomer with high oxygen permeability, has been introduced. It not only enhances oxygen transmission but also adsorbs moisture to generate hydroxyl groups, which helps alleviate dryness caused by increased hydrophobicity to some extent [12].

Besides oxygen permeability, the mechanical properties of CLs are also crucial in their design. The co-cross-linking of ethylene glycol dimethacrylate (EGDMA) and HEMA forms the foundation of modern soft CLs materials [13]. EGDMA chemically cross-links to the bis-hydroxyethyl structure of the HEMA monomer through the bis-methacrylate functional group in its molecular structure. This cross-linking significantly enhances the mechanical strength of the hydrogel, providing it with sufficient stiffness and abrasion resistance to withstand stretching and twisting during wear [14,15]. Although these cross-linked materials typically exhibit excellent oxygen permeability, they are often associated with a high elastic modulus.

A higher elastic modulus brings some advantages, such as facilitating the handling of CLs and improving the correction of corneal astigmatism. However, it may also be linked to various mechanical complications similar to those in silicone hydrogel lenses, including conjunctivitis and epithelial splitting [16,17,18,19]. In this context, polyethylene glycol diacrylate (PEGDA) has emerged as an ideal cross-linking agent, offering a promising solution. PEGDA is a derivative of polyethylene glycol (PEG), where the hydroxyl group at the end of the PEG chain is replaced by an acrylate group. PEGDA exhibits excellent hydrophilicity, good biocompatibility, and adjustable mechanical properties, making it an ideal cross-linker for manufacturing CL hydrogel materials. After adding the PEGDA monomer, the mixture is irradiated with ultraviolet (UV) light. This activates the photoinitiator molecules, leading to the absorption of light energy and the generation of free radicals. These free radicals then react with the acrylate groups at the ends of the PEG chains, forming covalent bonds between the chains and establishing a three-dimensional cross-linked polymer network. As a result, the hydrogel gains mechanical strength and stability [20,21,22]. Moreover, the high-strain and tensile modulus of polymers are generally determined by the length and molecular weight of the polymer chains [1]. Compared with low-molecular-weight PEGDA, high-molecular-weight PEGDA shows greater scalability in the cross-linked network and maintains long-term stability. High-molecular-weight PEGDA is also more effective in reducing adverse tissue reactions and immune responses, making it particularly promising in biomedical fields such as tissue engineering and drug delivery [23,24,25,26].

In this study, we mixed EGDMA with PEGDA with a molecular weight of 1000 Da, and introduced other silicone monomers (such as TRIS and KH-570) and hydrophilic monomers (such as HEMA and NVP) to prepare four samples with different formulations. By adjusting the weight percentages of each component, we optimized the optical properties and elastic modulus of the materials. The long-chain structure of PEGDA played a crucial role in this research. It increased the distance between cross-linking points, allowing polymer chains to move more freely. Consequently, the elastic modulus of the material was effectively reduced, and the flexibility was enhanced. This not only helped to reduce eye complications caused by a high elastic modulus but also improved the wearing comfort. Meanwhile, its hydrophilicity contributed to increasing the equilibrium water content (EWC) of the material, enhancing the wettability of the lens, and alleviating eye dryness issues. On the other hand, the presence of EGDMA ensured that the material had a certain mechanical strength, enabling CLs to maintain a stable shape and structure during the wearing process.

To achieve an optimal balance of optical performance, we adjusted the weight percentages of each component and measured the elastic modulus of the materials using an Electronic Universal Testing machine. We characterized the surface morphology using Atomic Force Microscopy (AFM) and Scanning Electron Microscopy (SEM). Fourier Transform Infrared Spectroscopy (FTIR) was employed to analyze the silicone hydrogel structure and determine the degree of curing. Additionally, we measured the EWC and transmittance of the samples. All of these results showed that hydrogels containing only PEGDA, without EGDMA, may exhibit similar curing rate, water content, and elastic modulus, but the hydrogels containing only PEGDA have poor optical transparency compared to hydrogels prepared with a mixture of PEGDA and EGDMA. When the ratio of PEGDA to EGDMA reached an optimal balance, the combination of both components enhanced the optical properties of the hydrogels. Furthermore, we simulated the imaging performance of the CLs using Ansys Zemax OpticStudio2024 software and evaluated the Modulation Transfer Function (MTF) and spot pattern, which provided effective support for assessing the visual quality of the CLs.

## 2. Materials and Methods

### 2.1. Material

3-(methacryloyloxy) propyltris (trimethylsiloxy) silane (TRIS), gamma-methacryloxypropyltrimethoxy silane (KH-570), ethylene glycol dimethacrylate (EGDMA), (2,4,6-trimethylbenzoyl) diphenylphosphine oxide (TPO), and 1-hydroxycyclohexyl phenyl ketone (Irgacure 184) were purchased from J&K Scientific (Beijing, China). Phosphate buffered saline solution (PBS, pH = 7.5), 2-hydroxyethyl methacrylate (HEMA), polyethylene glycol diacrylate (PEGDA), and 1-Hexanol solvent were purchased from J&K Scientific (Beijing, China). 1-vinyl-2-pyrrolidone (NVP) was purchased from J&K Scientific (Beijing, China) and Aladdin (Shanghai, China). All the compounds and solvents were used as received.

### 2.2. Preparation of CLs

**Figure 2:**
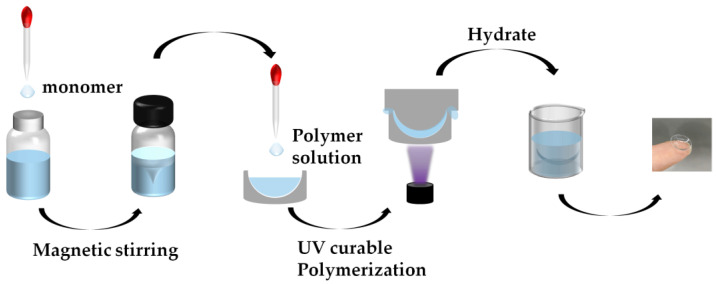
Schematic of fabrication of hydrogel CLs.

Figure 2 depicts the process of CLs preparation. Initially, Irgacure 184 and TPO were added to a brown sample bottle (shielded from light) in a 1:1 ratio. Subsequently, the silicone monomers TRIS and the KH-570 silane coupling agent, the hydrophilic monomers HEMA and NVP, as well as the cross-linking agents PEGDA and EGDMA, were added stepwise to the mixture. After that, the mixture was stirred using a magnetic stirrer for 5 h at room temperature in the dark. The liquid mixture was then transferred into CL molds and irradiated for 30–45 min using a 365 nm wavelength ultraviolet (UV) lamp with an intensity of 5 mW/cm^2^, resulting in the formation of a cross-linked network structure. Once the reaction was completed, the hydrogels were soaked in ethanol for 24 h to remove unreacted monomers. Then, they were immersed in deionized water to remove the ethanol, purify the hydrogel lenses, and finally stored in PBS at pH 7.5.

During the preliminary pre-experiments, we adjusted the contents of PEGDA and EGDMA multiple times. Through numerous trials, we discovered that when the content of PEGDA exceeded 20 wt%, the cured lenses would turn white noticeably, severely affecting the optical transparency. When the content of EGDMA exceeded 5 wt%, the lenses became brittle and failed to achieve satisfactory flexibility. The weight percentages of the final formulation were determined after multiple experiments and a comprehensive consideration of various properties. Table 1 provides details on the components and ratios of the synthesized hydrogels. In all the formulations used, the 1-hexanol solvent concentration was 5 wt%, the TPO concentration was 2 wt%, and the Irgacure 184 concentration was 2 wt%.

During the photocuring process, the light source activates the photoinitiator, generating free radicals that initiate the polymerization of monomers. However, the presence of oxygen can react with these free radicals to form less reactive peroxide species (such as peroxy radicals), which consume some of the free radicals and thus, slow down or inhibit the polymerization process. This phenomenon is known as oxygen inhibition. Acrylate monomers, in particular, are highly sensitive to oxygen inhibition. Therefore, it is essential to ensure the complete removal of oxygen from the solution before the cross-linking reaction starts to guarantee efficient polymerization. To mitigate the effects of oxygen inhibition, an adequate amount of photoinitiator is added to generate sufficient free radicals, compensating for the oxygen consumption [27].

### 2.3. Material and Membrane Characterization Techniques

#### 2.3.1. Fourier Transform Infrared Spectroscopy (FTIR)

Real-time infrared measurements of the hydrogel lenses were conducted using an FTIR spectrometer (Nicolet IS50 FTIR, Thermo Fisher Scientific, Madison, WI, USA) within the wavelength range of 500–4000 cm^−1^. During the FTIR measurement process, infrared spectra of the samples were taken at one-minute intervals for a total duration of 30 min. Moreover, parallel measurements were carried out three times at three different locations on the same sample. FTIR measurements can also be used to determine the conversion rate of double bonds, which represents the curing rate of the material after polymerization. This conversion rate was calculated using the following equation [28,29]:

(1) $$C\mathrm{onversion}\;\left(\%\right)=(1\;-\frac{M^{\prime }/R^{\prime }}{M/R})\;\times \;100$$

where $M^{\prime }/R^{\prime }$ represents the ratio of the measured peak to the reference peak area of the hydrogel after UV curing, and M/R stands for the ratio of the measured peak to the reference peak area of the hydrogel before UV curing.

#### 2.3.2. Mechanical Properties

To evaluate the mechanical properties of the hydrated lens, the elastic modulus was measured using an electronic universal testing machine (UTM6102, Shenzhen Suns Technology Stock Co., Ltd., Shenzhen, China) at a cross-head speed of 60 mm/min. Subsequently, the hydrated silicone hydrogels were cut into a dumbbell shape with a length of 36.36 mm, a middle width of 5 mm, and end widths of 9.95 mm.

#### 2.3.3. Equilibrium Water Content (EWC) and Equilibrium Weight Swelling Ratio

The EWC of the sample was calculated using the following formula:

(2) $$\mathrm{EWC}\;\left(\%\right)=\frac{W_{\mathrm{wet}}-W_{\mathrm{dry}}}{W_{\mathrm{wet}}}\;\times \;100$$

Herein, $W_{\mathrm{wet}}$ refers to the weight of the rehydrated sample after it has been immersed in deionized water at room temperature for 24 h, and $W_{\mathrm{dry}}$ is the weight of the sample after being dried in an oven for 12 h.

#### 2.3.4. Optical Transparency

The optical transparency of the samples was assessed based on their transmittance (T%). First of all, the lenses were immersed in deionized water. Subsequently, they were cut into rectangular pieces with dimensions of 1 cm × 1 cm. Thereafter, transmittance measurements were carried out using a double-beam UV-visible spectrophotometer (TU-1901 from PUXI, Beijing, China) within the wavelength range of 400–700 nm [30,31]. To ensure accuracy, each sample was scanned three times, and the average value was then calculated.

#### 2.3.5. Refractive Index and Abbe Number

The refractive index was measured with an ellipsometer (UVUSEL PLUS, Horiba, Kyoto, Japan). Meanwhile, the Abbe number was measured using an Abbe refractometer at wavelengths of 486.1 nm, 587.56 nm, and 656.3 nm. The Abbe number was then calculated using the following formula:

(3) $$v_{d}=\frac{(n_{d}-\;1)}{\left(n_{f}-{\;n}_{c}\right)}$$

where $v_{d}$ represents the Abbe number and $n_{c}$, $n_{d},$ and $n_{f}$ denote the refractive indices of the films at 486.1, 587.56, and 656.3 nm, respectively.

#### 2.3.6. Characterization of Silicone Hydrogel Lenses

An AFM (NT-DMT, NTEGRA, Moscow, Russia) and a SEM (GeminiSEM 560 from Zeiss, Oberkochen, Germany) were used to examine the surface morphology of the samples.

## 3. Results and Discussion

### 3.1. Synthesis of Silicone Hydrogels

**Figure 3:**
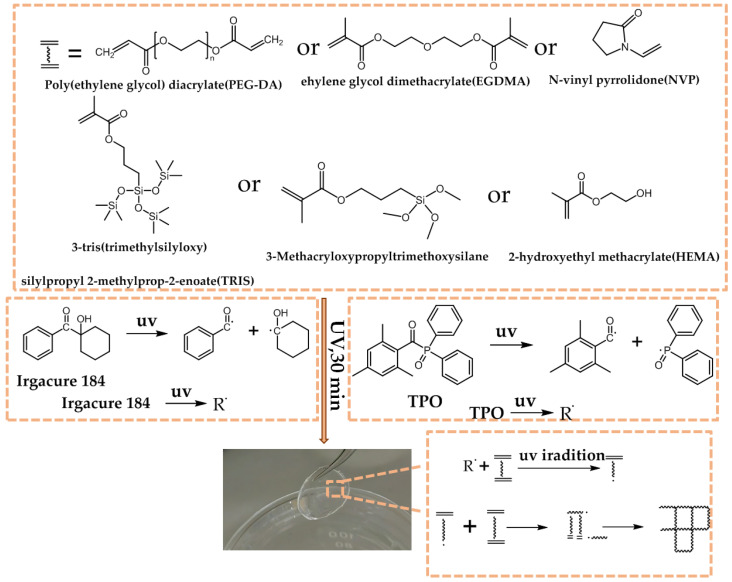
The photopolymerization process of CLs material.

Figure 3 depicts the photopolymerization process of organosilicon hydrogel CLs. In the process of preparing silicone hydrogel CLs, Irgacure 184 and TPO decompose under UV light, thereby generating reactive radicals, such as benzoyl radicals, cyclohexanol radicals, 2,4,6-trimethylbenzoyl radicals, and diphenylphosphinyl radicals. Moreover, the monomers involved in this process are HEMA, NVP, TRIS, the coupling agent KH-570, as well as the long-chain cross-linker PEGDA and the short-chain EGDMA. All of these monomers contain C=C bonds, which can serve as active centers and react with the free radicals cleaved from the photoinitiators to form new polymerization units. During the reaction, the newly formed monomer units keep participating in the polymerization and cross-linking reactions. Through the continuous repetition of the chain reaction, a hydrogel with a cross-linked network structure is ultimately formed [1,32].

### 3.2. FTIR Measurements and Curing Rate

**Figure 4:**
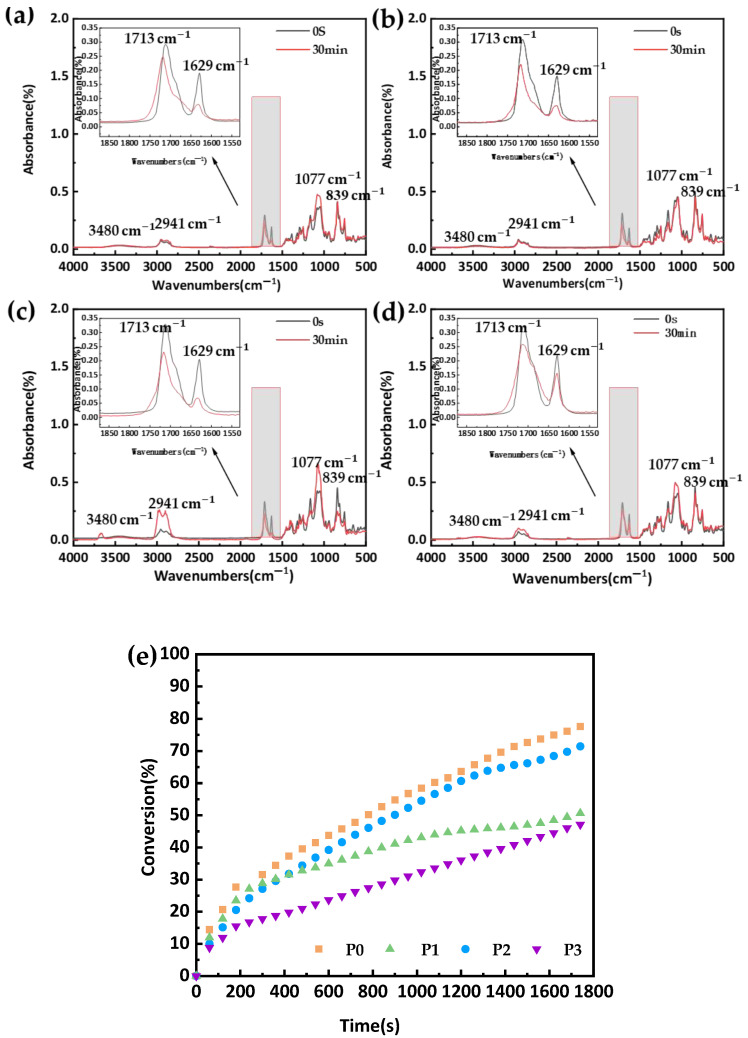
FTIR and double-bond conversion of the CLs material: (**a**) P0; (**b**) P1; (**c**) P2; (**d**) P3; (**e**) double bond conversion.

FTIR is one of the primary methods for studying the structure of materials after free radical polymerization. Figure 4a–d, respectively, present the infrared spectra of the P0, P1, P2, and P3 materials in their liquid states at 0 s and after 30 min of curing. Upon examination, we can observe only slight differences in the FTIR spectra of the four formulations. The major absorption peaks include the stretching vibration peak of the O-H bond at 3480 cm^−1^, the peaks of the Si-O-Si group of TRIS at 1077 cm^−1^ and 839 cm^−1^, and the antisymmetric stretching vibration peak of the C-H bond at 2941 cm^−1^. Additionally, the carbonyl C=O stretching vibration shows a significant absorption peak at 1712 cm^−1^, while the stretching vibration of the C=C double bond is also evident at 1629 cm^−1^. When comparing the absorption infrared spectra in the liquid state and after 30 min of UV curing, it is clear that there is a significant decrease in the absorption peak of the C=C double bond. This indicates that the opening of the double bond indeed occurred. This result is similar to the experimental results of some light-cured resin materials [29].

The quantitative analysis of the infrared spectrum is based on the measurement of the peak area of the characteristic absorption band to calculate the content of each component [33], and the theory is derived from the Lambert–Beer law [34]. Here, the relative peak ratio method was adopted: the infrared spectra of uncured raw materials and cured samples were both measured. The Origin 2019b software was used to integrate the selected measurement peak and reference peak which were integrated to calculate the area, and the curing rate was then obtained according to Equation (1). The C=C bond at 1629 cm^−1^ was chosen as the measurement peak because during the ultraviolet light-curing process, the C=C bonds of the material polymerize and react to form C-C bonds. The C=O bond at 1710 cm^−1^ was used as the reference peak because the C=O bonds of the material do not participate in the curing reaction. According to the C=C bond conversion rates of the four formulations shown in Figure 4e, the double-bond conversion rate of the material ranges from 45 to 75%. During the light-curing process, the double-bond conversion rate increases significantly as the curing time increases from 0 to 200 s, but the increase slows down after 200 s of curing. Referring to Table 1, the results in Figure 4 suggest that the incorporation of PEGDA may promote the conversion of C=C double bonds by extending the polymer chain length. Longer polymer chains facilitate polymer formation and may accelerate the conversion of double bonds during the reaction.

### 3.3. Ophthalmic Characterization

#### 3.3.1. The Mechanical Properties

**Figure 5:**
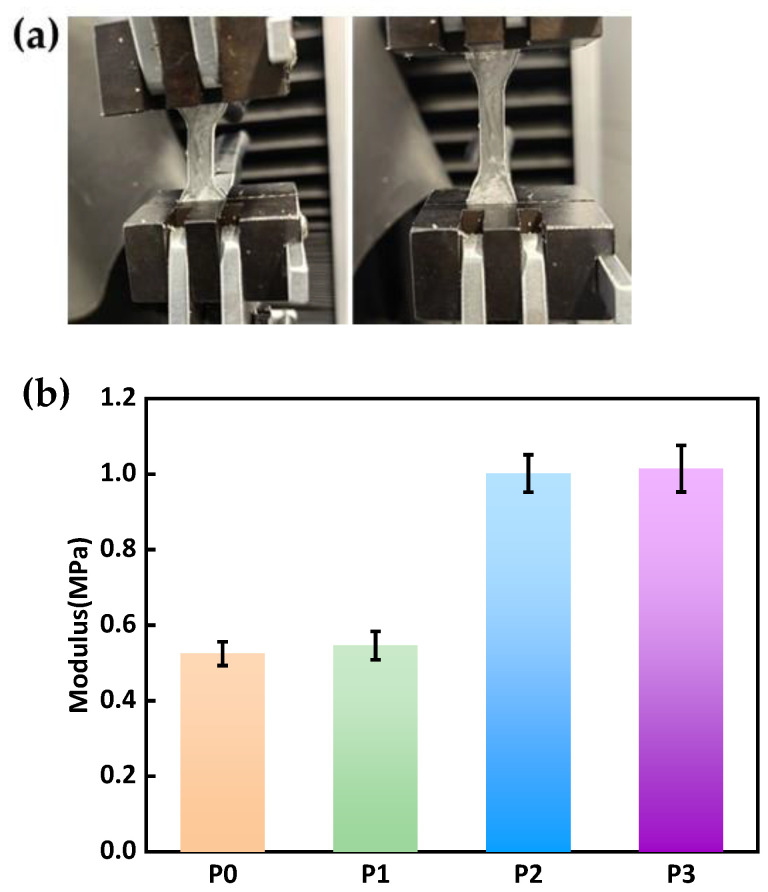
Mechanical testing of the CLs material: (**a**) the samples before and while in tension; (**b**) elastic modulus.

Studies have demonstrated that materials with a low elastic modulus offer a more comfortable wearing experience for contact lens users [35]. Figure 5a illustrates the state of the optical material before and after stretching. The elastic modulus data of the hydrogel material, which are derived from the experimental results, are presented in Figure 5b. By comparing the elastic modulus of different formulations in Table 1 and Figure 5b, it becomes clear that the elastic modulus of P0, P2, and P3 increase significantly as the PEGDA content decreases. Specifically, when the PEGDA monomer content drops from 18 to 10 wt%, the modulus of the lenses rises from 0.525 to 1.104 MPa, and all these values fall within the modulus range (0.43–1.52 MPa) required for commercial CLs [31,36]. This trend can be ascribed to the longer side-chain structure of PEGDA molecules. This structure increases the distance between cross-linking points [37]. Moreover, longer-chain cross-linkers are more flexible because they can rotate more freely around their ether bonds. The presence of multiple ether bonds enhances the material’s flexibility, enabling the polymer chains to migrate more freely, thereby reducing the hardness of the sample. The greater mobility of long-chain PEGDA monomers facilitates the sliding and movement between chain segments, making the material softer.

The P1 formula does not incorporate the short-chain EGDMA. The cross-linking agent in P1 is solely the long-chain PEGDA with a weight ratio of 14%. However, its elastic modulus can reach 0.546 MPa, and its performance is even superior to that of the P2 and P3 formulas. The reason for this is that the addition of short-chain cross-linkers increases the number of cross-linking points in the polymer, making the material hard, brittle, and prone to breakage, while long-chain cross-linkers do not have this issue.

The results of this study indicate that although the incorporation of long-chain PEGDA enhances the material’s flexibility, it still endows the material with a certain level of mechanical strength and toughness. These excellent mechanical properties enable the material to maintain its structural integrity during volume changes when absorbing water, effectively adsorbing and storing moisture without breaking or deforming. According to Tarnveer Singh Bhamra, research has shown that a modulus around 0.4 MPa is statistically the most popular value for current CLs material [36], so in this study, P0 (containing 18% PEGDA and 3% EGDMA) exhibits the best mechanical properties among the synthesized hydrogels.

#### 3.3.2. Equilibrium Water Content (EWC)

**Figure 6:**
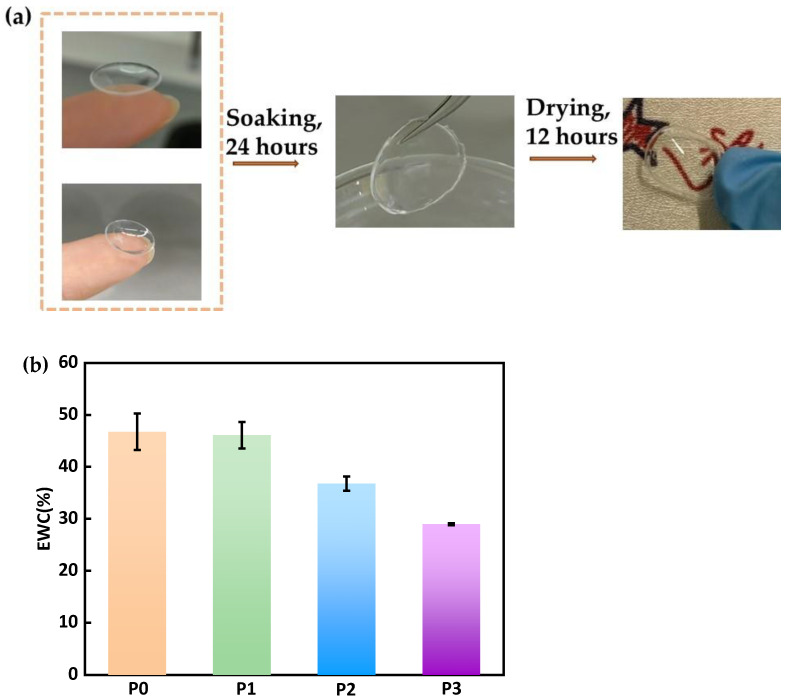
(**a**) Photos of Hydrated and dried CLs; (**b**) EWC of the CLs material.

Lens dehydration can cause dry eyes. Meanwhile, EWC, a crucial characteristic of CLs, is closely associated with corneal wettability. All four different formulations of the contact lens materials investigated in this study demonstrated high water absorbency due to the incorporation of various hydrophilic monomers. Figure 6a displays images of both hydrated and dried CLs, and Figure 6b presents the EWC calculated using Formula (2). It was found that a decrease in the PEGDA content led to a reduction in the EWC of the soft CLs from 46.735 to 28.95%. In comparison, commercial silicone hydrogel CLs typically have an EWC ranging from 24 to 56% [35,38]. The primary reason for this phenomenon is that long-chain PEGDA monomers contain multiple ethylene glycol units. These units possess strong hydrophilic properties and enhance the interaction between the material and water molecules. A higher PEGDA content increases the number of hydrophilic groups, thus improving the overall hydrophilicity of the material and making it more water-absorbent.

#### 3.3.3. The Correlation of Elastic Modulus, EWC, and Refractive Index

In the research field of CLs materials, in-depth analysis of the elastic modulus and EWC reveals that CLs with excellent water-absorption performance often have a lower elastic modulus [39,40]. Digging deeper into the underlying cause, this is highly likely due to the enhanced flexibility of polymer chains following water adsorption. A linear regression analysis of the elastic modulus and EWC has yielded the subsequent correlation equation:

(4) $$E\mathrm{lastic}\;M\mathrm{odulus}=-4.823\;\times \;\mathrm{EWC}+2.774\;{\;R}^{2}=0.999$$

Evidently, there exists an extremely strong linear relationship between the elastic modulus and EWC within the silicone hydrogel system.

The refractive index of soft CLs, being a precisely measurable key parameter, holds significant importance from both the vantage points of optical principles and human physiological adaptation. Generally speaking, within the silicone hydrogel material system, the refractive index and the elastic modulus display a positive-correlation tendency. Leveraging the data in Table 1, we were able to compute the linear regression equation and the correlation coefficient between them, and the outcomes are as follows:

(5) $$\mathrm{Refractive}\;\mathrm{Index}\;=0.086\;\times \;\mathrm{Elastic}\;\mathrm{Modulus}\;+2.774\;{\;R}^{2}=0.305$$

From this outcome, it is clearly discernible that the refractive indices of the three hydrated hydrogels, P0, P1, and P2, do increase as the elastic modulus rises. However, considering that the $R^{2}$ value is merely 0.305, this clearly suggests that apart from the elastic modulus, numerous other factors exert an influence on the refractive index. These potential factors encompass elements such as the chemical composition, molecular structure, and density of the material.

From a different perspective, the refractive index can also mirror the degree of the polymer’s EWC. According to the research findings of González-Méijome, in silicone hydrogel materials, the refractive index diminishes as the EWC increases [41]. Similarly, based on the data in Table 1, the calculated linear regression equation and correlation coefficient are:

(6) $$R\mathrm{efractive}\;I\mathrm{ndex}=-0.435\;\times \;\mathrm{EWC}+1.677\;{\;R}^{2}=0.322$$

This result indicates that the refractive indices of the P0, P1, and P2 hydrated hydrogels indeed decrease as the EWC increases. Nevertheless, since the R^2^ value is 0.322, it also implies that there are numerous other factors at work, such as the material type, additives, and preparation processes.

Nonetheless, a meticulous examination of the data in Table 1 reveals that the elastic modulus and EWC of the P3 sample exhibit distinct trends. We hypothesize that this is most probably closely associated with the substantial reduction in the PEGDA content. The significant decline in the PEGDA content might foster the formation of a denser cross-linked network within the material, thereby resulting in an increase in the elastic modulus and a decrease in the water content. However, the refractive index does not show a notable increase.

#### 3.3.4. Optical Properties

**Figure 7:**
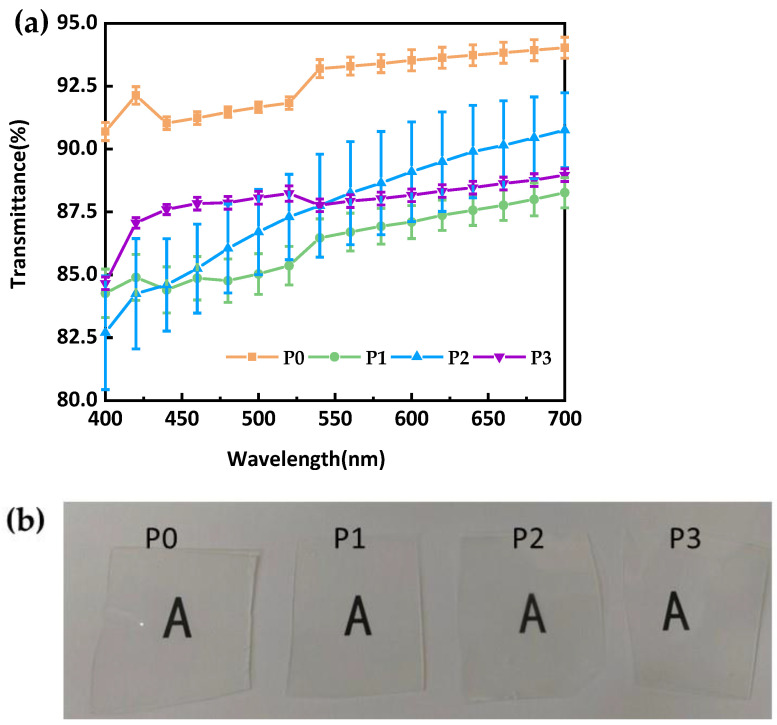
(**a**) Optical transmittance of CLs materials; (**b**) Photographs of synthesized flat samples.

Transparency is of utmost importance for the quality of CLs since it directly influences both the user’s visual experience and ocular health. As depicted in Figure 7a, we compared the light transmittance of four different formulations of silicone hydrogel lenses. Industry standards typically regard a light transmittance of over 85% as acceptable for contact lens materials [38]. Based on this criterion, lenses formulated with P0 showed excellent transparency, with their light transmittance exceeding 90% across all cases. This high transmittance allows the wearer to enjoy a clear and natural visual experience during use. It effectively mitigates the issue of blurred vision caused by insufficient light transmission, thus significantly enhancing the overall visual comfort. However, as the PEGDA content decreased, the transparency of the lenses started to decline remarkably, as evidenced by the P1, P2, and P3 formulations. Notably, in the 400 nm wavelength range, the transmittance of lenses made from the P1, P2, and P3 formulations was less than 85%, which is lower than the typical level of commercially available products. This implies that a portion of the light fails to reach the eye through the lens. Consequently, the eyes have to work harder to adapt to the visual environment, making it prone to causing uncomfortable symptoms like eye fatigue and dryness over an extended period.

After conducting multiple tests, we obtained results with a larger error range for sample P2 compared to other samples. We surmise that during the sample preparation process, the P2 sample was contaminated to some degree, which led to an increase in measurement error. This is attributed to the preparation process rather than the material itself. In the 400–550 nm wavelength range, the transmittance of the P2 lenses was significantly lower than that of the P3 lenses. It is worth noting that beyond the green-light wavelength range, the transmittance trends reversed. Although the P1 sample, which contains only PEGDA, exhibits good elastic modulus and water-absorption properties, Figure 7 clearly shows that this formulation has the lowest light transmittance, being significantly inferior to the silicone hydrogel material composed of a mixture of PEGDA and EGDMA. Figure 7b illustrates the light transmittance of flat material samples fabricated with the four different formulations, and all samples displayed good transparency performance.

The optical transparency of a material is primarily evaluated by the Abbe number. According to Table 1, the variation in Abbe numbers among these materials is minimal, indicating that they exhibit similar dispersion properties within the visible-light range and fall within the typical Abbe number range (40–50) of commercially available silicone hydrogel CLs [36]. Generally, silicone hydrogel materials with higher Abbe numbers tend to have lower dispersion, thereby providing better transparency and reduced chromatic aberration. However, in the four formulations investigated in this study, no significant correlation was observed between the Abbe number and light transmittance. This could be attributed to the presence of an incompletely removed photoinitiator in the formulation, which affects light absorption, or to the more prominent influence of the cross-linking structure on optical transparency.

### 3.4. Morphological Properties

**Figure 8:**
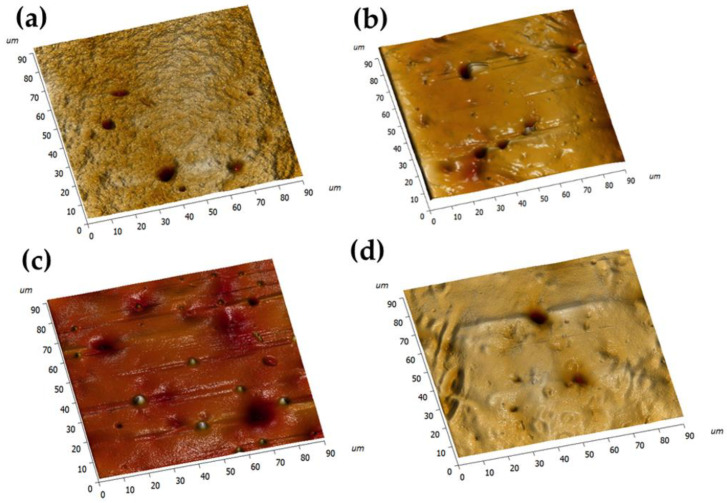
The AFM images of (**a**) P0; (**b**) P1; (**c**) P2; (**d**) P3.

In this study, we employed the tapping mode of an AFM to observe the morphology of the hydrogel. Figure 8 showcases AFM images of hydrated CLs captured from a 90 μm × 90 μm region. These images clearly expose typical pore and groove structures on the surface of the fabricated CLs materials [42]. Current research suggests that free chains of the hydrophilic PEG moiety are anticipated to emerge on the surface. This phenomenon potentially leads to a surface that is rougher yet more hydrophilic [43,44]. As the content of PEGDA rises, more voids emerge on the material surface, thus increasing the surface roughness. Concurrently, the formation of these voids also gives rise to an increase in the material’s water content. Our analysis results align with previous findings regarding water content.

**Figure 9:**
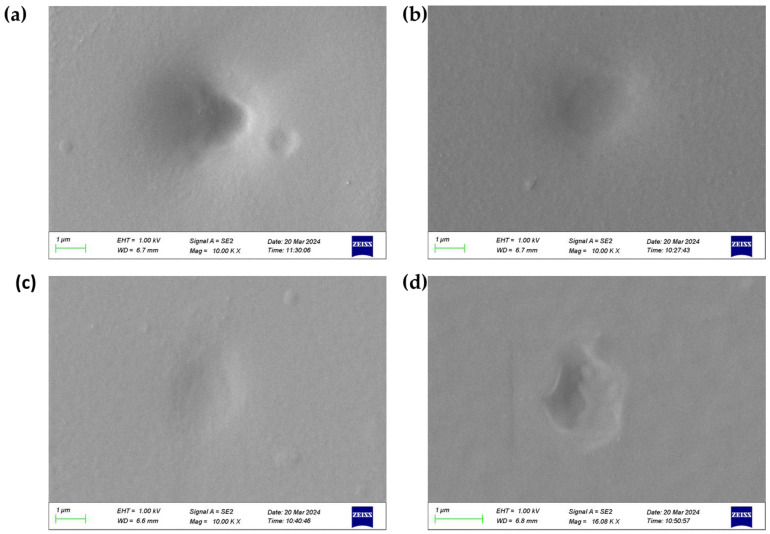
The SEM topographies of (**a**) P0; (**b**) P1; (**c**) P2; (**d**) P3.

For SEM analysis, dry samples are required to prevent artifacts like shrinkage or distortion during imaging. Thus, liquid nitrogen freeze-drying was selected as a method to maintain the structural integrity of the swollen thin films before SEM examination. Figure 9 presents the results of this processing step, revealing clear and consistent groove structures across the material surfaces. These findings confirm the observations made via AFM, further validating the presence of characteristic surface features on the materials under study.

### 3.5. Zemax Simulation Results

The Liou and Brennan eye (LBE) [45] model chosen for this study is an anatomically precise finite-eye model, which is extensively utilized in optical modeling. This model integrates detailed parameters of various ocular structures, as presented in Table 2. These parameters include the shape, curvature, and other characteristics of the cornea, lens, retina, and so forth, facilitating a more realistic simulation of the optical properties of the human eye. Based on the foregoing analysis, the P0 formulation strikes a favorable balance among key performance indicators such as elastic modulus, equilibrium water content, and optical transparency. This makes it a better embodiment of the desired properties for the CLs material in this research. Consequently, the P0 formulation was selected, and the Ansys Zemax OpticStudio2024 was employed for in-depth simulation analysis. The P0 CLs formulation (*n* = 1.4450, Abbe number $v_{d}$ = 47.89) was input into the software. Subsequently, the simulation results of the human eye distortion with these CLs incorporated were analyzed.

**Figure 10:**
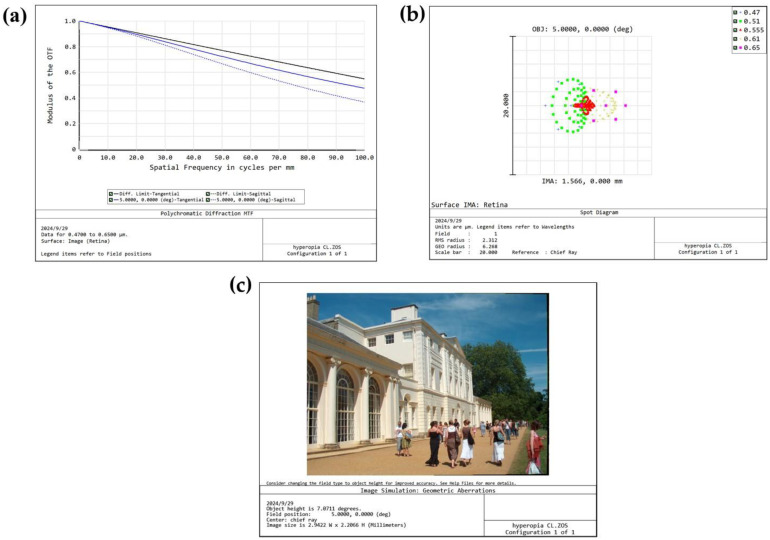
(**a**) MTF; (**b**) spot diagram; and (**c**) human eye retinal image simulation after adding CL.

The front-surface radius of the prepared contact lens was set at 8.5 mm, and its rear-surface radius was the same as that of the cornea in the LBE model, with a thickness of 0.1 mm. The simulation results are depicted in Figure 10. It can be observed that the resulting optical Modulation Transfer Function (MTF) curve after vision correction exhibited no significant aberrations, with a contrast exceeding 0.5 in the low-frequency range (20 cycles/mm). This indicates the ability to represent details distinguishable by the human eye [35]. The spot diagram shows an RMS value of 2.312 μm, demonstrating excellent correction efficacy. Finally, the retinal image simulation result reflected high imaging clarity, effectively validating both the MTF result and the point-spread function result. These results are comparable to those of Lina M. Shaker et al., [46] and Zahraa S. Alshaikhli et al. [47].

### 3.6. Research Limitation

While the mechanical properties and optical characteristics of proposed CLs have been comprehensively investigated, our current research still has certain limitations imposed by experimental conditions. Firstly, we could not precisely measure the oxygen permeability of the materials and the oxygen permeability is a crucial property of contact lens materials as it is directly linked to eye health. Secondly, due to the inability to comprehensively simulate the intricate physiological environment of the human eye, the research findings might not fully represent the actual usage scenario.

When CLs are worn, they are in direct contact with eye tissues and are influenced by numerous factors, including tear components, the ocular microbiota, and ocular immune responses. For instance, proteins, lipids, and enzymes in tears may adhere to the surface of CLs, thereby affecting their optical properties, wettability, and biocompatibility. Moreover, during actual use, CLs go through multiple processes such as wearing, cleaning, and storage, and these processes can cause the material properties to change over time. In the long-term use of CLs, materials may experience degradation and aging, which in turn impact their mechanical properties and biocompatibility. Owing to the lack of data from long-term stability tests, it is currently challenging to accurately forecast the performance changes in materials during long-term use, which may pose a certain obstacle to their practical applications.

## 4. Conclusions

We have successfully demonstrated the preparation of soft CLs with four different formulations (labeled as P0–P4). This was achieved by mixing the cross-linkers PEGDA and EGDMA and incorporating them into the silicone hydrogel. The photopolymerization and cross-linking processes of the monomers during UV light curing were analyzed and confirmed through FTIR measurements. It was found that the double-bond conversion rates for all four formulations exceeded 45% after 30 min, with the P0 formulation achieving a curing rate of over 70%. The key performance indicator parameters for CLs, including the elastic modulus, EWC, and light transmittance, were all measured and analyzed. As the PEGDA monomer content decreased from 18 to 10 wt%, the elastic modulus of the CLs increased from 0.525 to 1.104 MPa, while the EWC decreased from 46.735 to 28.95%. Additionally, the refractive index of the P0, P1, and P2 hydrogels decreased with both an increase in the elastic modulus and an increase in the EWC. All CLs with different formulations exhibited excellent light-transmission properties. Among them, P0 showed the best light transmission, with a transmittance of over 90%, while P1 had the worst. This difference might be attributed to the fact that the P0 sample was cross-linked with a mixture of PEGDA and EGDMA, whereas the P1 sample only contained PEGDA. Furthermore, the AFM and SEM analyses revealed typical porous and groove structures in all four formulations. The formation of voids in the materials also contributed to an increase in the EWC. Finally, the Zemax-simulated results indicated that the CLs with the P0 formulation could perform very well in the human-eye simulation model. Therefore, the optimum formulation proposed in this study has the potential to be applied in considerations for improving the performance and quality of CLs.

## Figures and Tables

**Table 1:** Compositions, refractive index (RI), Abbe number, equilibrium water content (EWC) and elastic modulus of CLs.

Samples	P0	P1	P2	P3
TRIS (wt%)	20	25	20	22
KH-570 (wt%)	20	25	20	22
HEMA (wt%)	15	16	19	17
NVP (wt%)	20	16	19	22
EGDMA (wt%)	3	0	3	3
PEGDA (wt%)	18	14	15	10
RI	1.4450	1.5164	1.5183	1.5152
Abbe number	47.89	48.26	47.99	47.70
EWC (%)	46.735 ± 3.494	46.100 ± 2.545	36.775 ± 1.364	28.950 ± 0.212
Modulus (MPa)	0.525 ± 0.031	0.546 ± 0.038	1.001 ± 0.049	1.104 ± 0.061

**Table 2:** Input parameters of the LBE model in Ansys Zemax OpticStudio2024. RI, refractive index; PD, pupil diameter; ${\;v}_{d}$, Abbe number.

Surface Type	Comment	Radius (mm)	Thickness (mm)	RI	$v_{d}$	Conic
OBJ standard	Object	Infinity	1000			0
1 Standard	Input Beam	Infinity	50			0
2 Standard	Cornea	7.77	0.55	1.383	50.23	−0.18
3 Standard	Aqueous	6.4	3.16	1.336	50.23	−0.6
4 Standard	Pupil	Infinity	0	1.336	50.23	0
5 Gradient 3	Lens-front	12.4	1.59			0
6 Gradient 3	Lens-back	Infinity	2.43			0
7 Standard	Vitreous	−8.1	16.24	1.336	50.23	0.96
IMG Standard	Retina	−12.0				0

## Data Availability

The original contributions presented in this study are included in the article. Further inquiries can be directed to the corresponding author.
